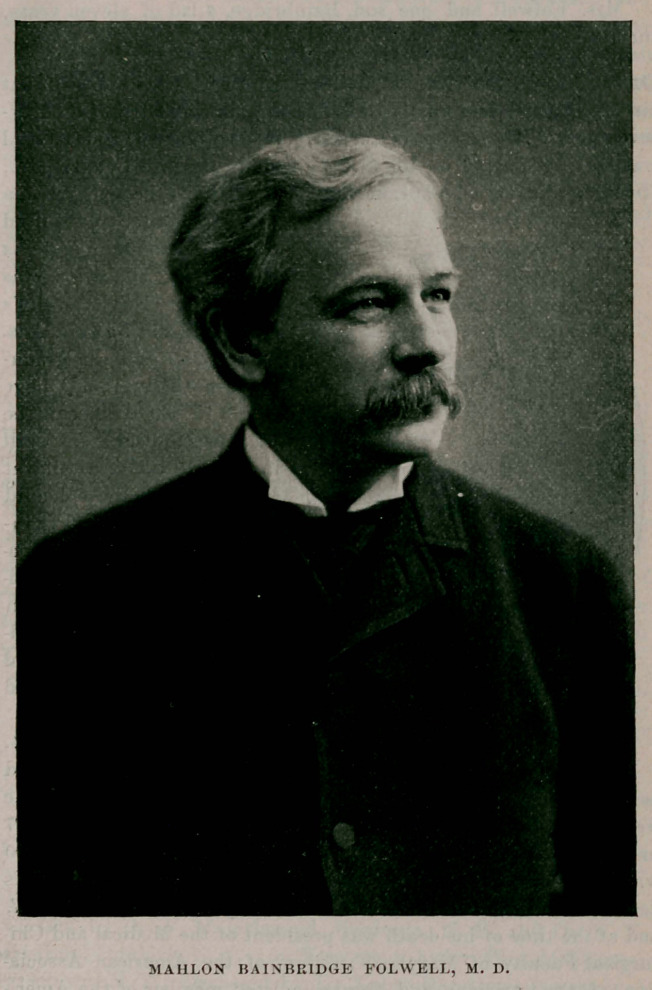# Mahlon Bainbridge Folwell, M. D.

**Published:** 1896-01

**Authors:** 


### Obituary.

### MAHLON BAINBRIDGE FOLWELL, M. D.

Dr. Mahlon Bainbridge Folwell died at his home, 713 Delaware avenue, Buffalo, Tuesday, December 10, 1895, of pericarditis, aged fifty-four years. He was born at Romulus, Seneca County, N. Y., in 1841 and received his academic education at Hobart College, Geneva, from which he graduated in 1861. After spending a year in the study of medicine he entered the army as hospital steward of the Fiftieth New York Engineers, April 10, 1862; was promoted first-lieutenant, Company I, May 19, 1863; captain, February 1, 1865, and was mustered out with his regiment June 13, 1865. He was made brevet-captain and major, U. S. Volunteers, August 1, 1864, for gallant and meritorious services during the campaign before Richmond, Va. He was at the siege of Yorktown, served in the Fredericksburg and Peninsular campaigns, was at Chancellorsville, Mine Run and in the Wilderness campaign, also at the siege of Petersburg and in the Appomattox campaign in the spring of 1865.

After the close of the war he came to Buffalo and pursued his medical studies under Dr. Wyckoff, receiving his doctorate from Buffalo University Medical College in 1867. He afterward became associated in practice with Dr. George N. Burwell and in December, 1882, Dr. Folwell married Florence, daughter of Leonidas Doty, of Buffalo. He was a consulting physician at Buffalo General Hospital; attending physician at the Buffalo Orphan Asylum and at the Children's Hospital; and was clinical professor of diseases of children at the medical department, University of Buffalo. Dr. Folwell was a member of the Medical Society of the County of Erie, Buffalo Academy of Medicine, Buffalo Medical Club, the Liberal, Buffalo, Saturn and University Clubs and a companion of the Military Order of the Loyal Legion.

In another place in the Journal Dr. Folwell's intimate personal and professional friends have testified in a fitting manner their opinions regarding his professional and social status and accomplishments. But it is appropriate for us to add that the estimate there given of his character and manliness is not overdrawn. Dr. Folwell was an ornament to the local profession of medicine, a physician of rare acumen and judgment who enjoyed

MAHLON BAINBRIDGE FOLWELL, M. D.

the confidence of a large clientèle, a man of sterling character and a citizen whose loss is deeply deplored by the entire community.

Mrs. Folwell and one son, Bainbridge, a lad of eleven years, survive.

## Figures and Tables

**Figure f1:**